# Molecular characterization and antibiotics resistance of *Aeromonas* species isolated from farmed African catfish *Clarias gariepinus* Burchell, 1822

**DOI:** 10.1186/s12917-023-03860-5

**Published:** 2024-01-06

**Authors:** Deborah Arimie Adah, Lawal Saidu, Sonnie Joshua Oniye, Adakole Sylvanus Adah, Oluwafemi Babatunde Daoudu, Shola David Ola-Fadunsin

**Affiliations:** 1 Department of Veterinary Medicine, Faculty of Veterinary Medicine, University of Ilorin, Ilorin, Nigeria; 2 Veterinary Teaching Hospital, Ahmadu Bello University, Zaria, Nigeria; 3 Department of Biological Science, National Open University of Nigeria, Abuja, Nigeria; 4 Department of Veterinary Physiology and Biochemistry, Faculty of Veterinary Medicine, University of Ilorin, Ilorin, Nigeria; 5 Department of Veterinary Microbiology, Faculty of Veterinary Medicine, University of Ilorin, Ilorin, Nigeria; 6 Department of Veterinary Parasitology and Entomology, Faculty of Veterinary Medicine, University of Ilorin, Ilorin, Nigeria

**Keywords:** Aquaculture, Phylogenetic identification, Antimicrobial resistance, 16S rRNA

## Abstract

**Background:**

*Aeromonas* species are one of the most important etiologies of diseases in fish farms, leading to clinical manifestation and mortality and are associated with public health risks. This study aimed to investigate the prevalence, phenotypic and molecular characteristics of *Aeromonas* species isolated from farmed *Clarias gariepinus* using 16 S rRNA sequencing. Additionally, their antibiogram and multiple antibiotic resistance index were determined using a disc diffusion test.

**Results:**

A total of 230 *Aeromonas* strains were isolated from *Clarias gariepinus* with 40.9% obtained from diseased fish, and 25% isolated from apparently healthy ones. Five different species including *Aeromonas caviae, Aeromonas veronii*, *Aeromonas hydrophila*, *Aeromonas dhakensis* and *Aeromonas enteropelogenes* were fully identified and genetically characterized. Based on the available literature, this is the first report of *Aeromonas enteropelogenes* from the study area. The phylogenetic analysis showed genetic heterogeneity and distance within the species and the reference strains. The multiple resistant *Aeromonas* species were susceptible to ciprofloxacin, gentamycin, and florfenicol. The *Aeromonas* species’ multiple antibiotic resistance index values varied between 0.20 and 0.80 and were isolated from the farms where antibiotics were intensively used.

**Conclusions:**

The diversity of multidrug-resistant *Aeromonas* species isolated from fish farms is a major threat to fish production giving us more understanding of epidemiology and the multidrug *Aeromonas* species with a MAR index of greater than 0.2 were isolated from farms where antibiotic use was widespread. As a result, a considerably increased danger of multiple antibiotic resistance spreading to the fish culture environment may impact aquaculture production. Hence there is a need for appropriate and monitored drug usage.

## Background

*Aeromonas* species are autochthonous microflora of aquatic environments and have been reported as a significant etiological agent of fish disease resulting in significant financial losses in the global aquaculture industry [1–4].

*Aeromonas* infection affects a wide range of fish species, among them is *Clarias gariepinus*. The infection is often characterized by hemorrhages on the skin and fins, ulcers, abdominal distension, exophthalmia, congestion and fin rot resulting in slowed growth, increases in morbidity and mortality on farms, as well as higher production costs, lower earnings, and food insecurity and more critically can be transmitted to humans [1, 2].

*Aeromonas* species are Gram-negative rod-shaped bacilli, oxidase and catalase positive [3, 4], and presently have more than 30 genospecies, according to recent taxonomy [5–7]. The routine identification of *Aeromonas* species in fish farms typically involves traditional methods such as microbiological culture and biochemical tests. However, these methods are recognized for their time-consuming nature and occasional inconsistencies in findings and diagnoses due to the significant diversity both between and within species. Consequently, molecular characterization is often favored as a more preferable choice [8–11]. Precise and rapid molecular based identification of *Aeromonas* species would be a useful diagnostic tool in clinical and veterinary laboratories and necessary for outbreak prediction, management, and control interventions in aquaculture [7, 12–14].

Multidrug resistance has escalated globally, posing a significant public health concern. Recent studies have documented the emergence of multidrug-resistant bacterial pathogens from diverse sources, underscoring the need for judicious antibiotic use. Additionally, routine antimicrobial susceptibility testing is crucial for identifying the appropriate antibiotics and screening for the presence of emerging multidrug-resistant strains [15–17].

In the developing world, fish mortality has been regarded as one of the major risks facing the aquaculture sector with *Aeromonas* species being the main etiology of fish mortality [18–21]. It is therefore imperative and crucial to comprehend the nature and types of *Aeromonas* species from the fish farm and their susceptibility patterns within the study area. Hence, this study aimed to isolate, identify, and molecularly characterize *Aeromonas* species from *Clarias gariepinus* from fish farms and determine their antibiotic susceptibility.

## Results

### Clinical signs and postmortem lesions

**Fig. 1:**
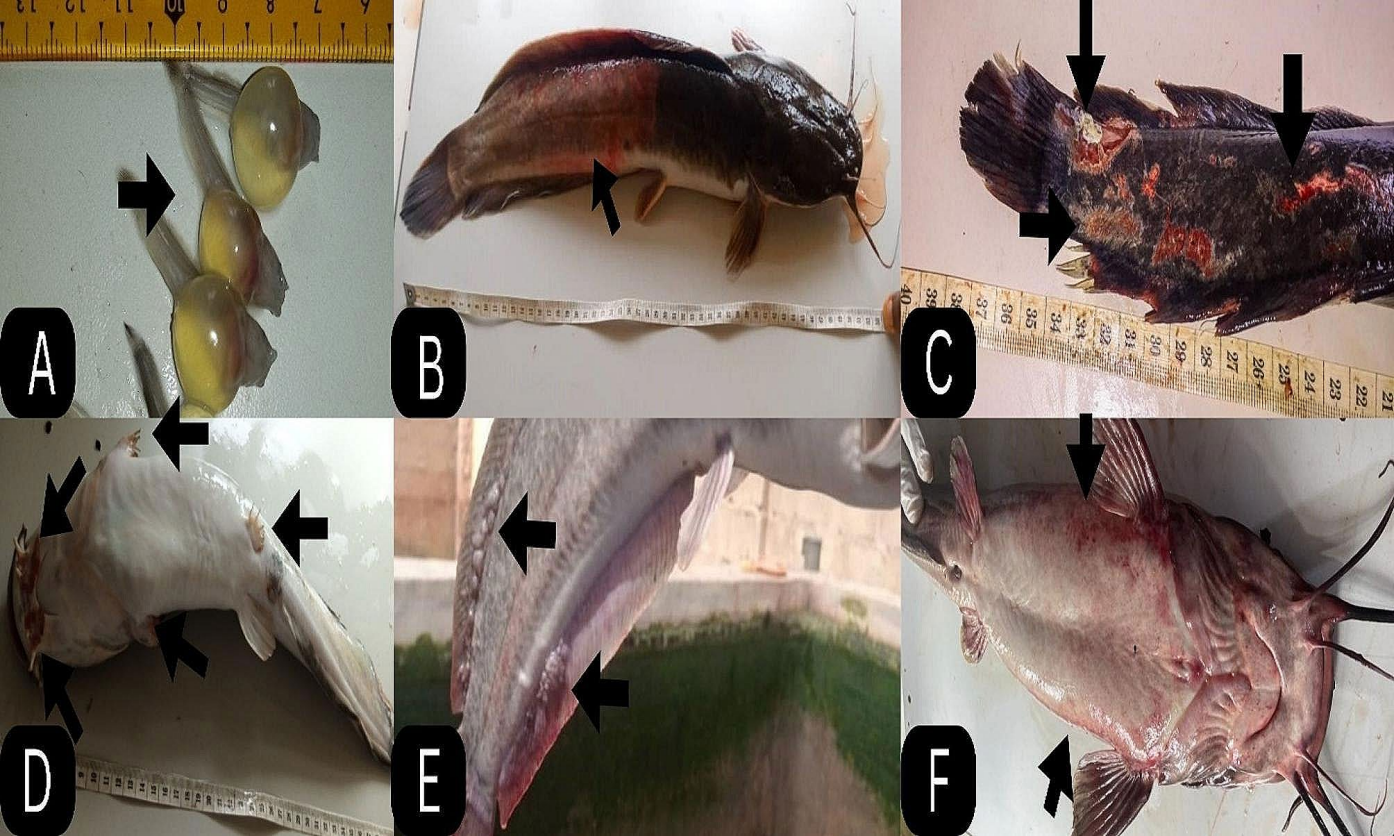
**(A-F)** Clinical signs associated with *Aeromonas* species isolated from *C. gariepinus*. **A**) Dropsy in fingerlings **B**) Hemorrhages on the skin **C**) Erosion and lesion on the skin. **D**: Degeneration of the barbel and fins **E**): Blister on the skin of the fish F: hemorrhages and discoloration of the skin

**Fig. 2:**
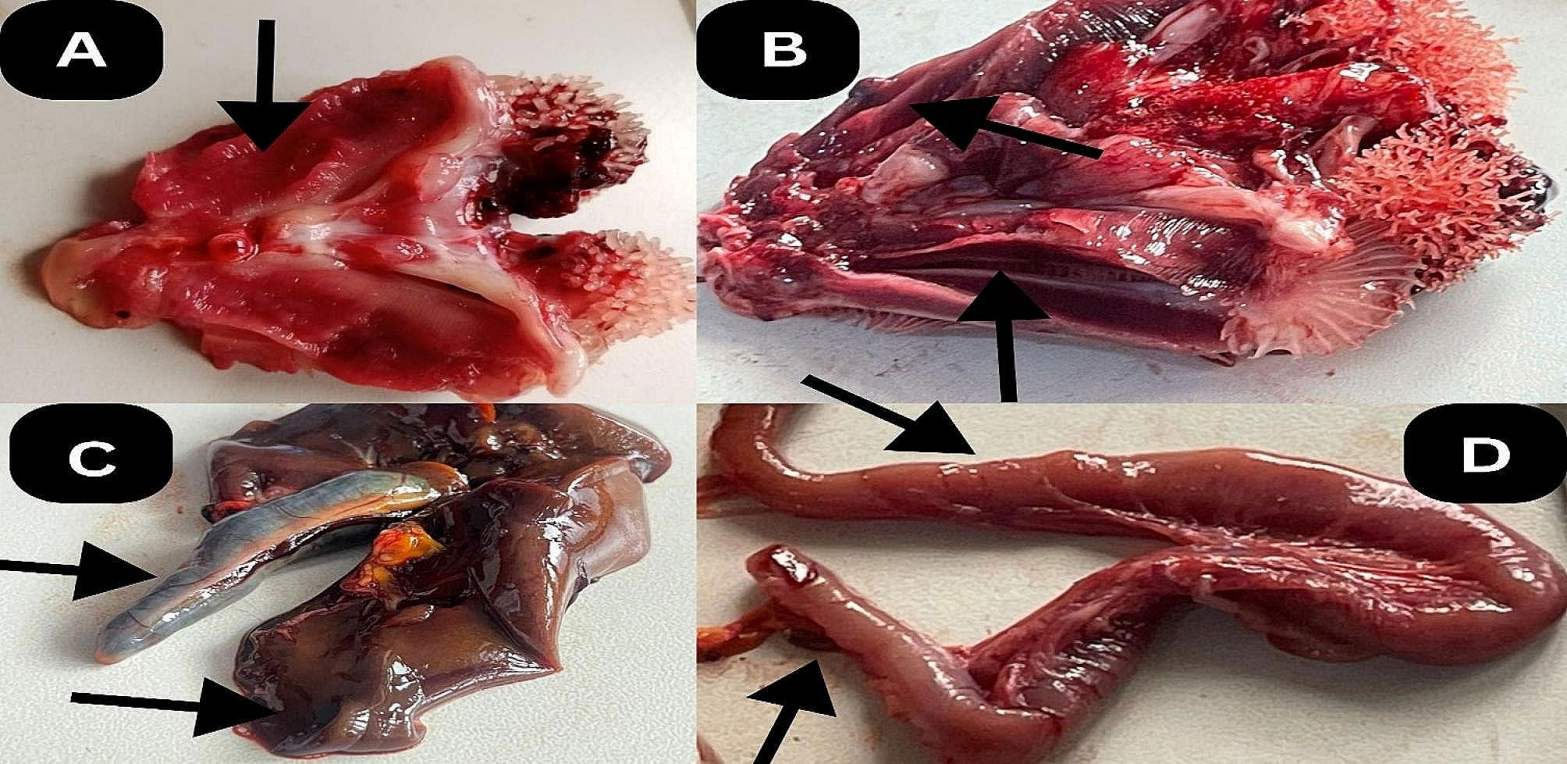
**(A-D)** Postmortem lesions associated with *Aeromonas* species isolated from *C. gariepinus***(A)** Haemorrhagic and inflamed gills **(B)** Hemorrhages of the gills **(C)** Congested kidneys, discoloration of the kidney and liver, enlarged liver, gall bladder. **D**: Ballooned, hemorrhagic and intestinal congestion

**Fig. 3:**
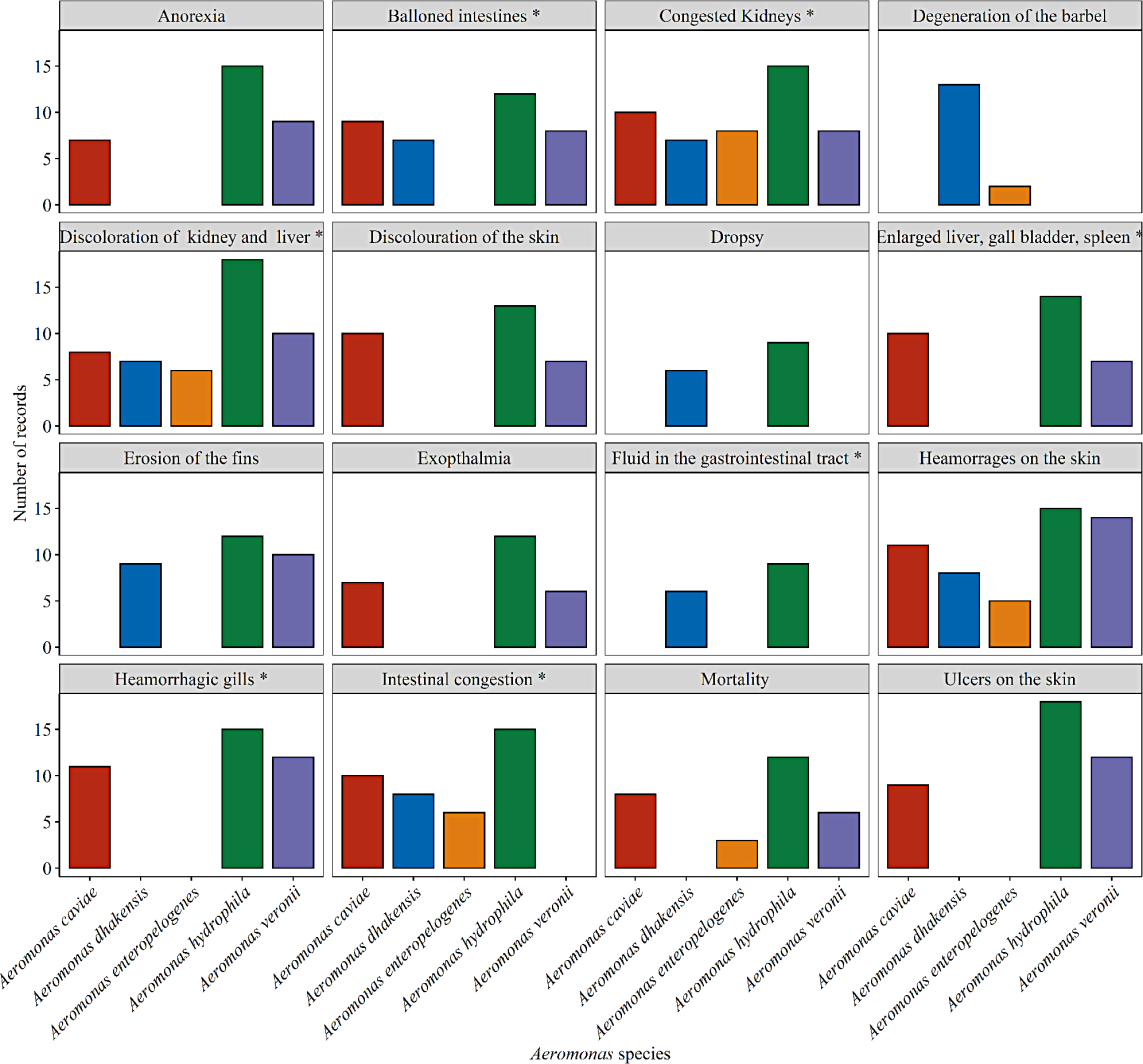
The frequency, severity and distribution of clinical signs and postmortem lesions associated with *Aeromonas* species. *: signifies postmortem lesions

The clinical signs observed in *C. gariepinus* in this study were characterized by anorexia, dropsy, degeneration of the barbel, discoloration of the skin, erosion of the fins, exophthalmia and hemorrhages on the skin (Fig. 1). The postmortem lesions observed were ballooned intestines, congested kidneys, discoloration of the kidney and liver, enlarged liver, gall bladder, spleen, fluid in the gastrointestinal tract, hemorrhagic gills and intestinal congestion (Fig. 2). The frequency, severity and distribution of clinical signs and postmortem lesions associated with *Aeromonas* species from the infected fish revealed that most of the signs were observed in *A. hydrophila* (94%) followed by *A. caviae* and *A. veronii* (75%) then *A. dhakensis* (50%) and the least was *A. enteropelogenes*. The clinical signs differed significantly between the *Aeromonas* species isolated at *P* < 0.05 (Fig. 3).

### Prevalence of *Aeromonas* species from *Clarias gariepinus* from the study area

The results of biochemical identification using the Microbact 24E Kit demonstrated that all species were Gram-negative, motile, and rod-shaped and showed characteristics of *Aeromonas* species to be *A. caviae*, *A. dhakensis*, *A. enteropelogenes*, *A. hydrophila* and *A. veronii*. Subsequently, the isolates were confirmed by molecular characterization using 16S rRNA-PCR. A total of two hundred and thirty *Aeromonas* species were isolated from *C*. *gariepinus* with 40.9% obtained from diseased *C. gariepinus* while 25% was isolated from apparently healthy *C. gariepinus. A. caviae* was the most prevalent species isolated from diseased fish followed by *A. hydrophila*, then *A. dhakensis* and the least prevalent was *A. enteropelogenes*. However, from the apparently healthy fish, the most prevalent species was *A. caviae* followed by *A. hydrophila*, then *A. veronii* then *A. dhakensis* and the least observed prevalence was for *A. enteropelogenes*. The prevalence of the *Aeromonas* species differed significantly between the healthy and diseased fish (Table 1). Two to five different *Aeromonas* species were found in the heterogeneous mixture isolated from the fish. Among the mixed species isolated from the fish farms three distinct species of *Aeromonas* were the most prevalent (Table 2).

**Table 1:** Prevalence of *Aeromonas* species among naturally infected (diseased) and apparent healthy *Clarias gariepinus*

*Aeromonas* species	Number infected (%) of the diseased *Clarias gariepinus* (440)	Number infected (%) of the healthy *Clarias gariepinus* (200)
*Aeromonas caviae*	63 (14.3) ^a^	17 (8.5) ^b^
*Aeromonas dhakensis*	18 (4.1) ^a^	5 (2.5) ^a^
*Aeromonas enteropelogenes*	9 (2.0) ^a^	3 (1.5) ^a^
*Aeromonas hydrophila*	37 (8.4) ^a^	15 (7.5) ^a^
*Aeromonas veronii*	53 (12.0) ^a^	10 (5.0) ^b^
**Total**	**180 (40.9)** ^**a**^	**50 (25.0)** ^**b**^

Different letters across rows indicate significance (*P* < 0.05)

**Table 2:** Prevalence of the mixed *Aeromonas* species infections that were noticed among the investigated fish

Clinical signs / Post mortem Lesions	N (%)	*Aeromonas* species isolated	Prevalence (%)
Dropsy	150 (23.4)	*Aeromonas hydrophila, Aeromonas dhakensis*	2 (12.5%)
Fluid in the gastrointestinal tract	150 (23.4)	*Aeromonas hydrophila, Aeromonas dhakensis*	2
Anorexia	375 (58.6)	*Aeromonas caviae, Aeromonas veronii, Aeromonas hydrophila*	3 (50%)
Degeneration of the barbel	280 (43.8)	*Aeromonas enteropelogenes, Aeromonas dhakensis*, *Aeromonas caviae*	3
Discoloration of the skin	300 (46.9)	*Aeromonas caviae, Aeromonas veronii, Aeromonas hydrophila*	3
Erosion of the fins	315 (49.2)	*Aeromonas hydrophila, Aeromonas dhakensis, Aeromonas veronii*,	3
Exophthalmia	250 (39.1)	*Aeromonas caviae, Aeromonas veronii, Aeromonas hydrophila*	3
Ulcers on the skin	320 (50.0)	*Aeromonas caviae, Aeromonas veronii, Aeromonas hydrophila*	3
Hemorrhagic gills	360 (56.3)	*Aeromonas caviae, Aeromonas veronii, Aeromonas hydrophila*	*3*
Enlarged liver, gall bladder, spleen	323 (50.5)	*Aeromonas caviae, Aeromonas veronii, Aeromonas hydrophila*	*3*
Ballooned intestines	370(57.8)	*Aeromonas caviae, Aeromonas veronii, Aeromonas hydrophila, Aeromonas dhakensis*	4 (12.5%)
Intestinal congestion	280 (43.8)	*Aeromonas enteropelogenes, Aeromonas dhakensis Aeromonas caviae, Aeromonas hydrophila*	4
Hemorrhages on the skin	420 (65.6)	*Aeromonas caviae, Aeromonas veronii, Aeromonas hydrophila, Aeromonas enteropelogenes, Aeromonas dhakensis*	5 (25%)
Congested Kidneys	400 (62.5)	*Aeromonas caviae, Aeromonas veronii, Aeromonas hydrophila, Aeromonas enteropelogenes, Aeromonas dhakensis*	5
Discoloration of the kidney and liver	370 (62.5)	*Aeromonas caviae, Aeromonas veronii, Aeromonas hydrophila, Aeromonas enteropelogenes, Aeromonas dhakensis*	5
Congested Kidneys	400 (62.5)	*Aeromonas caviae, Aeromonas veronii, Aeromonas hydrophila, Aeromonas enteropelogenes, Aeromonas dhakensis*	5

### Molecular characterization and phylogenetic analysis of *Aeromonas* strains

**Fig. 4:**
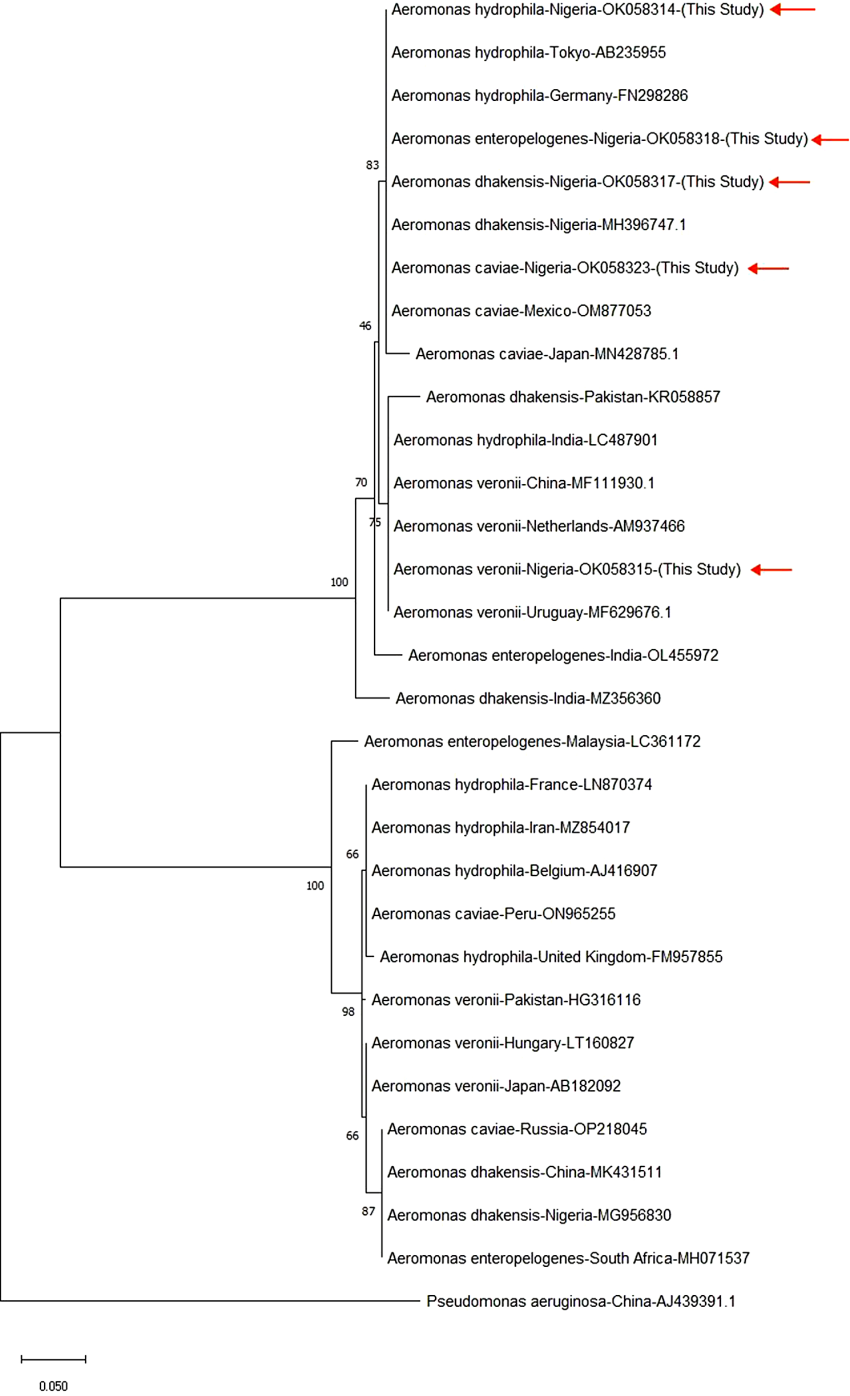
Phylogenetic tree constructed for *Aeromonas* species based on 16 S rRNA sequences

The amplification and sequencing of the 16 S rRNA of all *Aeromonas* species with the following accession numbers OK058314, OK058315, OK058317, OK058318 and OK058328 were carried out with nucleotide sequences ranging in similarity from 98 to 100% to that of the GenBank nucleotide sequence database. According to the branching pattern, all of our *Aeromonas* species are clearly divided into two major clades in the phylogenetic tree created by the neighbor-joining method using 16 S rRNA. The phylogenetic tree shows the genetic relationships between isolated and reference strains. Based on the geographical location of the isolates, the trees also displayed genetic heterogeneity and distance within the species (Fig. 4).

### Antibiotic resistance and multiple antibiotic index of *Aeromonas* species

**Fig. 5:**
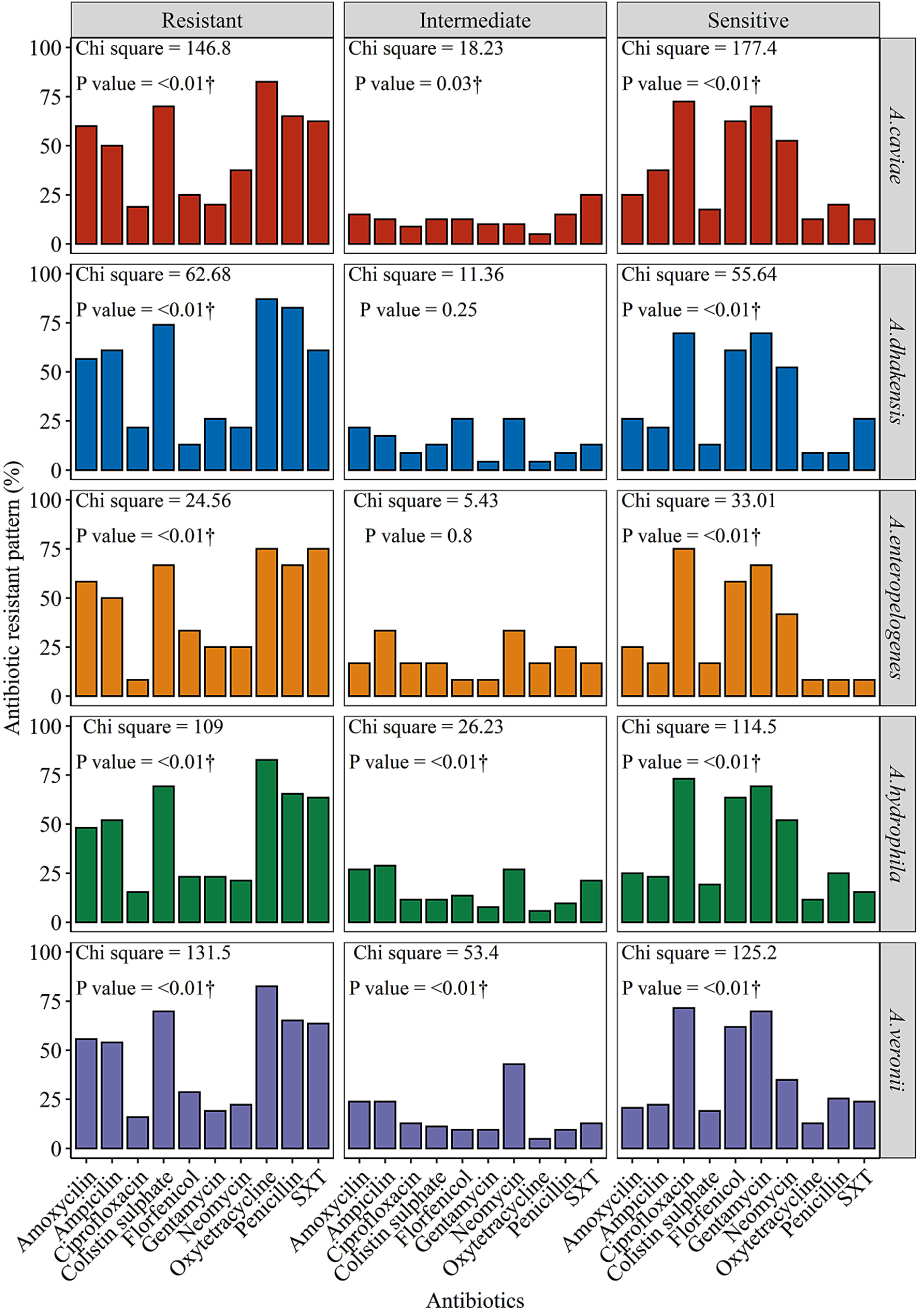
Distribution of antibiotic susceptibility among *Aeromonas* species to ten antimicrobials

The isolated *Aeromonas* species showed varying degrees of antimicrobial resistance to more than six of the antibiotics used. Oxytetracycline (82.5%) showed the highest resistance to *A. caviae*, followed by colistin sulfate (70.0%), penicillin (65.0%), trimethoprim/sulfamethoxazole **(**62.5%), amoxicillin (60.0%), and ampicillin (50.0%) also showed high levels of resistance. Ciprofloxacin (18.0%), gentamycin (20.0%), florfenicol (25%) and neomycin (35.0%) showed the lowest levels of resistance to *A. caviae*. Furthermore, there was a significant difference in resistance to the various antibiotics utilized (*P* < 0.01) for *A. caviae* (Fig. 5). *A. dhakensis* isolated in this study had a high resistance level to oxytetracycline (87.0%), followed by penicillin (82.6%), colistin sulfate (73.9%), ampicillin and trimethoprim/sulfamethoxazole (60.9%) respectively. More than 50% were resistant to amoxicillin, resistances to ciprofloxacin, gentamycin, neomycin and florfenicol were less than 26%, with the least resistance observed for florfenicol (13.0%), with significant difference *(P* < 0.01) observed to the antibiotics used (Fig. 5).

A similar antibiotic resistant pattern was recorded for *A. enteropelogenes* with the highest resistance recorded for oxytetracycline and trimethoprim**/**sulfamethoxazole (75.0%), followed by penicillin and colistin sulfate (66.7%), amoxicillin (58.3%) and ampicillin (50.0%). Minimal resistance was recorded for ciprofloxacin, gentamycin, neomycin and florfenicol, with the least resistance observed for ciprofloxacin (8.3%), the resistance observed differed significantly (*P* < 0.01) (Fig. 3). For *A. hydrophila* high antibiotic resistance to oxytetracycline (82.7%), colistin sulfate (69.2%), penicillin (65.4%), trimethoprim/sulfamethoxazole (63.5%), ampicillin (51.9%) and amoxicillin (48.1%) was observed. Approximately, 23.1% of the *A*. *hydrophila* recorded lower resistance for gentamycin and florfenicol (21.2%), neomycin (20.1%) and the lowest resistance of (15.4%) observed for ciprofloxacin there was a significant difference in the antibiotic used (*P* < 0.01) (Fig. 5).

Additionally, the isolated *A. veronii* also displayed high resistance to oxytetracycline (82.5%), penicillin (69.8%), colistin sulfate (65.1%), trimethoprim/sulfamethoxazole (63.5%), amoxicillin (55.6%) and to ampicillin (54.0%). About 28.6% of the *A. veronii* showed lower resistance to florfenicol, 22.2% to neomycin, 19.0% to gentamycin and the least resistance of 15.9% observed for ciprofloxacin, there was a significant difference in the antibiotic used (*P* < 0.01) (Fig. 5).

Despite having similar patterns of susceptibility to ciprofloxacin, gentamycin, and florfenicol within the *Aeromonas* species. There was also a significant variation (*P* < 0.01) in the susceptibility of the *Aeromonas* species to the antibiotics used.

### The association among antimicrobial resistance of the antibiotics

**Fig. 6:**
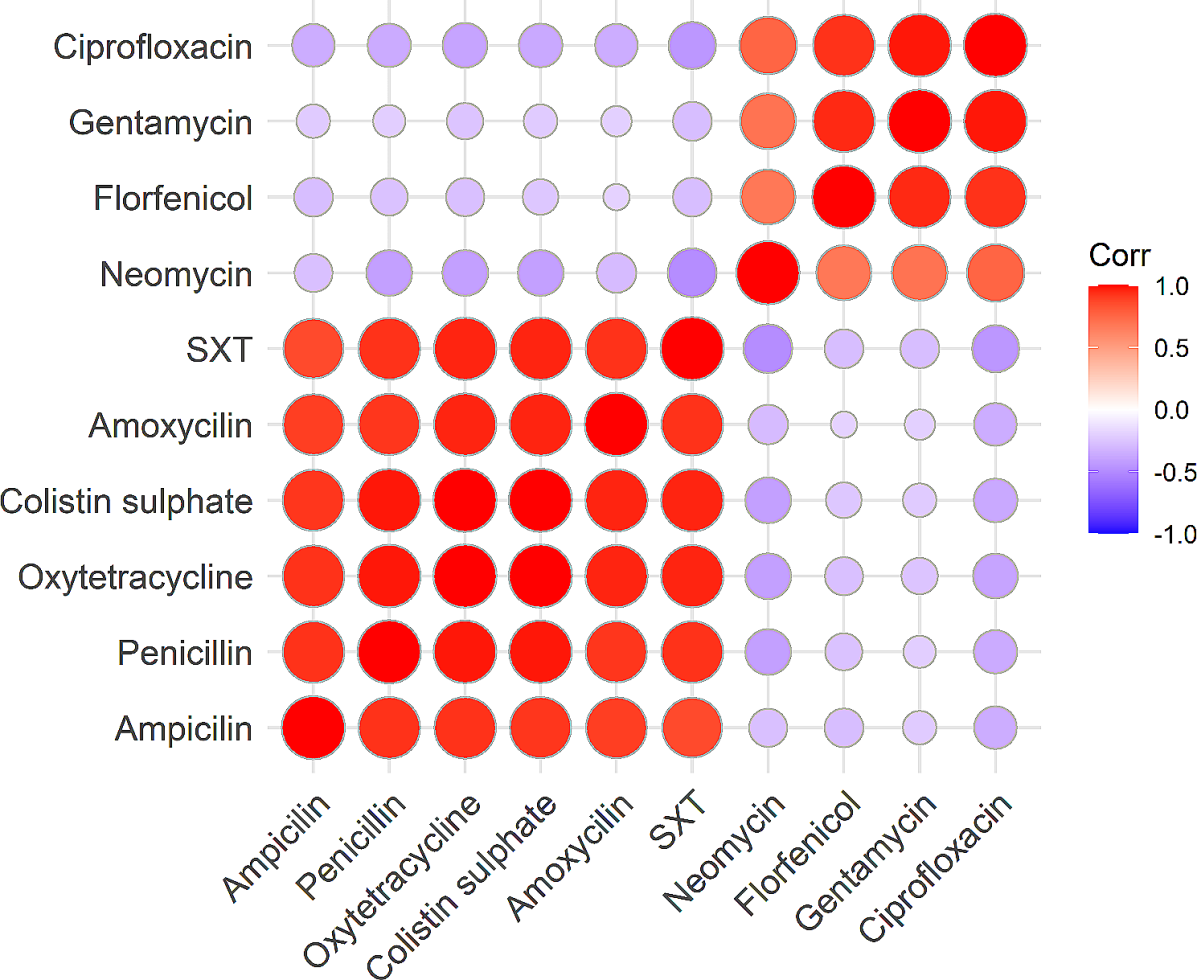
Shows the correlation (r) of antibiotic resistance; positive and negative correlations are denoted by the color red and purple respectively. SXT: Trimethoprim**/**sulfamethoxazole

**Fig. 7:**
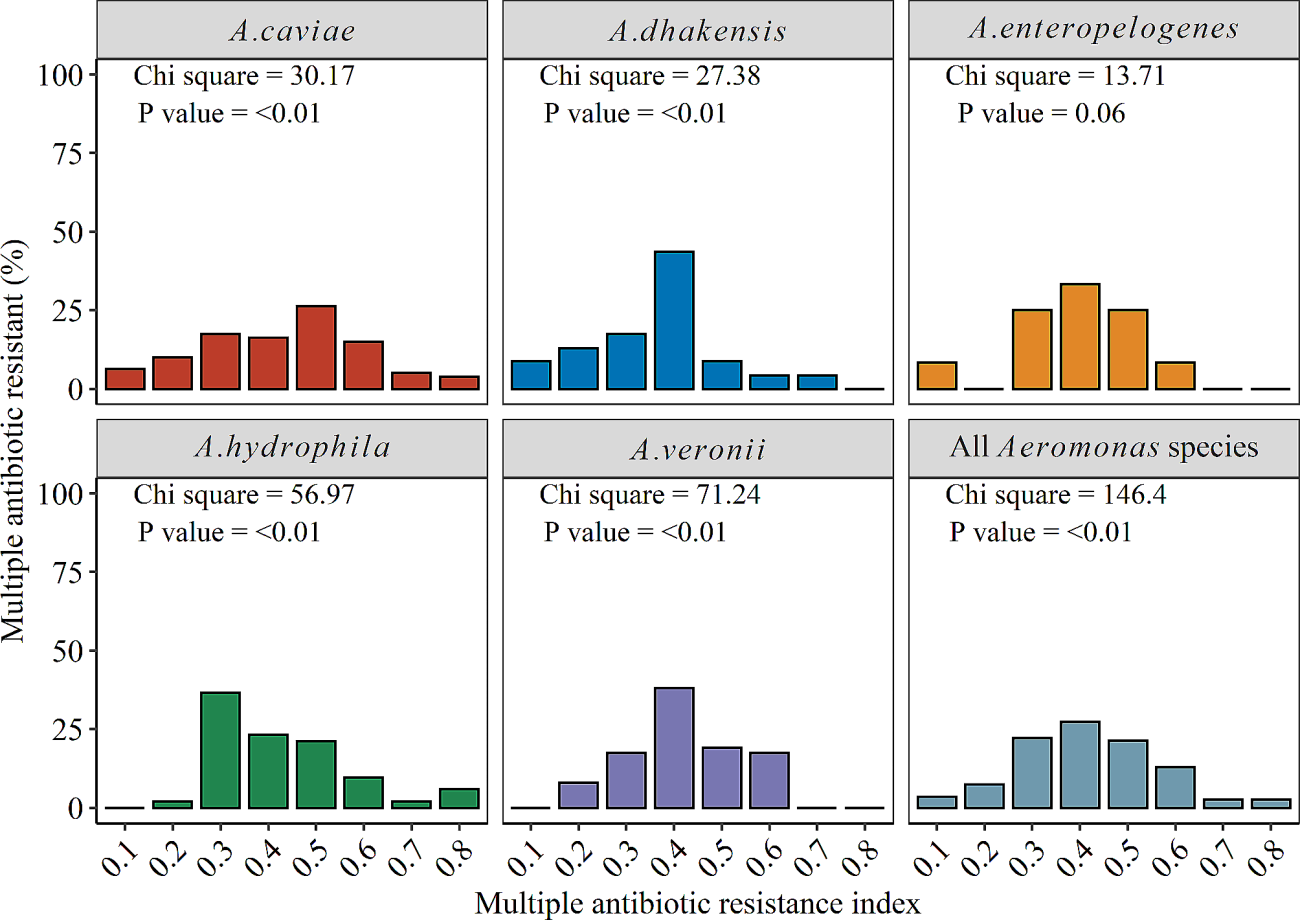
Distribution of the multiple antibiotic resistance index of Aeromonas species isolated in the study area

According to our findings, resistance to ampicillin was strongly and positively correlated with penicillin, oxytetracycline, colistin sulfate, trimethoprim/sulfamethoxazole and amoxicillin, but negatively and significantly correlated with ciprofloxacin, neomycin and florfenicol (Fig. 6). The multiple antibiotic resistance index of all the *Aeromonas* species ranged between 0.2 and 0.8 and varied significantly among the *Aeromonas* species (Fig. 7).

The classification of the resistant *Aeromonas* species revealed a notable difference between the extensive drug-resistant species and the multidrug-resistant species (Table 3).

**Table 3:** Classification of the multidrug resistant *Aeromonas* species isolated in this study

*Aeromonas* species	Multidrug-resistant (MDR) N (%)	Extensively drug-resistant (XDR) N (%)	Non resistant *Aeromonas* species N(%)
*Aeromonas caviae* (80)	35(43.75)	40(50.00)	5(6.25)
*Aeromonas dhakensis* (23)	9 (39.13)	12 (52.17)	2(8.70)
*Aeromonas enteropelogenes* (12)	3(25.00)	8 (66.67)	1(8.33)
*Aeromonas hydrophila* (52)	20(38.46)	32(61.54)	0
*Aeromonas veronii* (63)	16 (25.39)	47(74.60)	0
All *Aeromonas* species (230)	83 (36.09)	139 (60.43)	8(3.47)
*χ*^2^ value	174.1	310.3	19.48
*P* value	0.0001	0.0001	0.001

*χ*^2^ value : Chi square; N: Number

## Discussion

Disease outbreaks of *Aeromonas* species are one of the most destructive infectious diseases affecting farmed fish which poses serious production issues leading to mortality and great economic loss and is an important global mitigating factor for sustainable fish production owing to the ability of *Aeromonas* species to produce a variety of clinical manifestations in fish [1, 4]. The clinical signs observed in this study which manifested as erosions, hemorrhages and inflammation have been reported by Dias et al. [22], Adah et al. [2] and Marinho-Neto et al. [23] in that, the skin can be invaded by *Aeromonas* species which can also damage blood vessels and cause ulcerative lesions with a hemorrhagic appearance leading to inflammation as recorded in this study.

Isolation and identification of *Aeromonas* species from apparently healthy and diseased *C. gariepinus* in this study were similar to the reports of Omeje and Chukwu [19]; Mzula et al. [5], Adah et al. [2] and Dhanapala et al. [24]. As a result of this finding, when there are changes in management practices and fluctuations in the water quality of fish farms, the isolation of seemingly clinically healthy fish may cause an outbreak of disease leading to mortality and loss as observed in this study.

The prevalence of *Aeromonas* species from disease *C. gariepinus* was similar to the findings of Attia et al. [25] who isolated *Aeromonas* species from fish samples, however, it was higher than the reports of El-Gohary et al. [26]and Tartor et al. [27]and lower than the findings of (Abd-El-Malek, [28]; Elghareeb et al. [29]; Saengsitthisak et al. [30]. Different interactions between the pathogen, host, and environment may have led to the variation of the prevalence rates of the different *Aeromonas* species from both diseased and healthy fish have been reported by Ramesh and Souissi, [31] and Algammal et al. [32].

The most commonly isolated species are *A. hydrophila*, A. *caviae*, and A. *veronii.* This is similar to the reports of Saengsitthisak et al. [30], Dhanapala et al. [24] and De Silva et al. [33]. However, the frequency of distribution of the isolated *Aeromonas* species revealed that *A. caviae* was the most prevalent species followed by *A*. *veronii* and then *A*. *hydrophila*. This is in agreement with the findings of Ebeed et al. [34]; Fernández-Bravo and Figueras [35]; Mulia et al. [36]and Fauzi et al. [37]. However, this is contrary to the report that *A. hydrophila* [2, 4, 38, 39] and *A. veronii* [7, 40–42], were the most prevalent species. The isolation of the different species of *Aeromonas* from fish is an additional indication that these bacteria are geographically widespread and are associated with disease in fish farms around the world [32, 43]. To the best of our knowledge, this is the first report of *A. enteropelogenes* isolated from farmed *C. gariepinus* from the study area. *Aeromonas enteropelogenes* have been associated with disease outbreaks in freshwater ornamental fish [43] and in freshwater fish [31].

Disease outbreaks can only be effectively controlled if the etiological agent is accurately identified [31, 44] and one of the reliable molecular methods for the identification of *Aeromonas* species is 16 S rRNA sequencing [34, 35, 45, 46]. The capacity to differentiate between the isolates for which the biochemical identification was unsure in this study was made possible by 16 S rRNA sequencing, demonstrating the applicability and dependability of this molecular technique in identifying *Aeromonas* species [12, 41]. Our findings on the phylogenetic analysis of the isolated *Aeromonas* species were closely related to each other and closely related to *Aeromonas* species from Tokyo, Germany China, Mexico, Netherlands and Uruguay.

The *Aeromonas* species showed multiple antibiotic susceptibility patterns with significant resistance to β-lactam antibiotics (amoxicillin, ampicillin, and penicillin). Saengsitthisak et al. [30]; Nhinh et al. [4] and De Silva et al. [33] also reported a similar high resistance to these β-lactamases, which might be because several, inducible, chromosomally encoded beta-lactamases are produced. Additionally, the multi-antibiotic resistant *Aeromonas* species were also resistant to oxytetracycline, neomycin, sulfamethoxazole, and colistin sulfate, among other antibiotics [47–49]. This may be attributable to the widespread usage of these medications, which are easily obtained over the counter and administered via feeds or baths [39, 43]. This resistance observed has great consequences for both fish and human health, however, the *Aeromonas* species were susceptible to ciprofloxacin, gentamycin, and florfenicol, which is in agreement with the findings of Algammal et al. [42], Mazumder et al. [49] and Adah et al. [50]. These results might be explained by the fact that, in comparison to other antibiotics, these drugs are not used as frequently in aquaculture. This finding is however, in contrast with the results of Dhanapala et al. [24], El-Gohary et al. [26] and Lin et al. [51] reported that *Aeromonas* species were resistant to ciprofloxacin, gentamycin, and florfenicol.

The high level of multiple drug resistance in this study is of great concern to fish production and a public health risk [31]. The *Aeromonas* species had a high MAR index ranging between 0.20 and 0.80 which was similar to the findings of Dhanapala et al. [24] and El-Gohary et al. [26]. *Aeromonas* species isolated from fish culture environments have been reported to have a high MAR index, Hossain et al. [52] recorded a MAR of 0.19–0.44 from zebra fish; Preena et al. [47] observed a MAR index ranging between 0.04 and 0.46 from *Oreochromis niloticus* and Fauzi et al. [37] recorded a MAR index value of 0.07 to 0.64 from freshwater fish. MAR index greater than 0.2 indicates that the *Aeromonas* species from *C. gariepinus* may have been exposed to the uncontrolled use of antibiotics during culture, subsequently leading to the development and incidence of antibiotic resistance, negatively impacting the effectiveness of treatment in fish farms.

## Conclusions

From the results obtained it was concluded that there was a diversity of multidrug-resistant *Aeromonas* species isolated from the fish farms sampled following the biochemical and molecular techniques carried out in this study, giving us more understanding of bacterial identification prevalence and epidemiology. This is the first report of *A. enteropelogenes* in the study area. The multidrug *Aeromonas* species with a MAR index of greater than 0.2 were isolated from farms where antibiotic use was widespread. As a result, there may be a considerably increased danger of MAR spreading to the fish culture environment, which may impact aquaculture production. As a result, regular monitoring and the use of antimicrobial susceptibility tests and appropriate antibiotic usage are needed.

## Methods

### Fish sample collection

Six hundred and forty *C. gariepinus* weighing between 50 g and 1.3 kg and a total length of 10 -46 cm were sampled from thirty-six active fish farms, of earthen ponds, plastic and concrete tanks over the period of May 2019 to April 2020. *Clarias gariepinus* in various stages of development (fingerlings, growers, adults, and brood stocks) were stocked on the fish farms. Four hundred and forty diseased and two hundred healthy fish were randomly collected alive from the farms and transported in plastic receptacles containing water from the culture facility to the fish clinic of the Veterinary Teaching Hospital University of Ilorin for further diagnostic procedures and examination (Table 4).

**Table 4:** Distribution of the Fish farms and fish samples collected

Culture facility	Number of Fish farms	Farm management style	Average number of fish sample/ Farm	Diseased Fish	Apparently healthy fish	Total number of fish sampled	Average weight (g)	Average total length (cm)
Concrete Ponds	9	Intensive System	22–24	146	67	213	150-1200 g	20–40 cm
Earthen Pond	9	Semi intensive System	22–24	148	66	214	200-1300 g	25-46 cm
Plastic Tanks	9	Intensive System	22–24	146	67	213	50-1000 g	10-35 cm

### Clinical and postmortem examination of the fish samples

The sampling technique was carried out in accordance with the standards for fish disease diagnosis and aquatic animal health monitoring [53]. All the samples of the fish obtained were evaluated clinically and a postmortem examination was carried out as described by Austin [54]. Each live fish was euthanized by placing the fish in the water from the fish farms then 300 mg/L buffered tricaine methanesulfonate (MS-222) Syncaine® USA was added and left there for at least 10 min. After the cessation of opercular movement, the fish was removed and pithed before the fish were dissected. Samples from the skin, gills, gastrointestinal tract, kidney liver and spleen were collected aseptically as described by Austin [54].

### Bacterial isolation and identification

Based on observation on the fish farms, bacteria were isolated from both diseased and apparently healthy fish. Portions of the skin, gills, liver, heart, kidney, GIT, and spleen were weighed aseptically and placed in separate labeled Kryo bottles containing 20 mL of alkaline peptone water (Oxoid Basingstoke, England. United Kingdom) as the pre-enrichment broth and incubated at 37 °C for 24 h. Growth in the selective enrichment cultures was transferred with a sterile loop, inoculated onto selective *Aeromonas* agar (Oxoid Basingstoke, England. United Kingdom) supplemented with ampicillin (10 mg/L) and incubated at 37 ^°^ C for 24 h, after which dark green, opaque with dark centers colonies were presumptive for *Aeromonas* species were streaked on MacConkeys agar (MCA) (Oxoid Basingstoke, England. United Kingdom) plates and incubated at 37^°^ C for 24 h. Gram reaction, oxidase and catalase tests were performed [54].

### Biochemical identification

All suspected presumptive colonies were collected and examined for phenotypic and biochemical characteristics. The biochemical characterization of the *Aeromonas* isolates was assessed by conventional biochemical tests such as the citrate test, hydrogen sulfide, indole test, methyl red test, motility test, sugar (glucose, inositol, and mannitol) urease test, Voges Proskauer test [55], and confirmed using Oxoid rapid microbat identification test kits for Gram-negative bacteria, Microbact 24E (MB24E) (Oxoid Ltd, Basingstoke, England. United Kingdom). Additionally, the evaluation of Gram staining, colonial features, motility, oxidase, and catalase activities, as well as growth on various agar and in response to various temperature conditions, was determined [53, 56].

### Molecular characterization and phylogenetic analysis of *Aeromonas* species

The DNA was extracted using the Quick-DNA Fungi/Bacterial Miniprep Kit with catalog number D6005 from Zymo Research (ZR) following the manufacturer’s instructions to confirm the presumptively identified *Aeromonas* species. Prior to the molecular analysis, the concentration and purity of all DNA samples were optimized by DNA electrophoresis. The 16S rRNA was amplified using the conventional polymerase chain reactions (PCR) and was used to characterize the *Aeromonas* isolates to species level. The nucleotide sequences and specifications of 16S F (5’GTGCCAGCAGCCGCGCTAA3’) and 16 S R:(5’AGACCCGGGAACGTATTCAC3’), synthesized at Inqaba Biotech South Africa were used for this study [57]. The primer sequences, PCR amplification, and sequencing were performed in accordance with previous reports [58]. DNA sequence data was determined using GenBank database searches and BLAST programs at the National Center for Biotechnology Institute (NCBI) (http://blast.st-va.ncbi.nlm.nih.gov/Blast. cgi). Furthermore, the nucleotide sequences were submitted to GenBank BLAST. A search for each isolate was conducted with the sequences generated for each isolate on the NCBI database, which gave the identities of each isolate. Finally, the acquired sequences were then modified using Bio Edit version 7.0. [59]. After this, the MEGA 7.0 program was used to create a phylogenetic tree using the neighbor-joining method (1000 bootstraps) in which the two-parameter Kimura method was used to calculate the evolutionary distances [60].

### Antibiotic susceptibility and multiple antibiotic resistance (MAR) index

The resistance of the identified *Aeromonas* species to the ten commonly used antibiotics including amoxicillin (30 µg), ampicillin (10 µg), ciprofloxacin (5 µg), colistin sulphate (10 µg), florfenicol (30 µg), gentamycin (10 µg), neomycin (30 µg) oxytetracycline (30 µg), penicillin (10 IU ) and trimethoprim/sulfamethoxazole (SXT) (25 µg ) (Oxoid, Ltd, Basingstoke, England. United Kingdom) was determined by the standard disc diffusion method according to the guidelines of the Clinical and Laboratory Standards Institute [61]. By measuring the diameter of the zones (in mm) around the disc, antibiotics were interpreted according to the CLSI criteria [61]. The multiple antibiotic resistance (MAR) index was determined as the proportion of resistant phenotypes to all the antibiotics to which the bacteria were exposed [24]. As previously reported by Magiorakos, et al., [62] the examined isolates are classified as extensively drug-resistant (XDR), pan drug-resistant (PDR), and multidrug-resistant (MDR).

### Statistical analysis

A Microsoft Excel 2016 spreadsheet was used to first enter all of the data gathered from this study. The Statistical Package for the Social Sciences for Windows version 20.0 was used to conduct the statistical analysis to determine the prevalence rates of the *Aeromonas* species. Additionally, the percentage of *Aeromonas* species resistance was also determined for each antibiotic and the degree of resistance for each antibiotic was compared using the Chi-squared test. To visualize the antimicrobial resistance phenotypes of the isolated *Aeromonas* species a heatmap was generated and correlated. Using the R package version 4.0.5 for the Windows system, correlation analysis was carried out as described previously by Galili et al. [63]. Values of *P* < 0.05 with a 95% confidence interval were considered significant.

## Data Availability

The datasets generated and analyzed during the current study are available in the NCBI GenBank repository, under the accession numbers: OK058314, OK058315, OK058317, OK058318 and OK058328.

## Abbreviations

BLAST: Basic local alignment search tool
CLSI: Clinical and laboratory standards institute
DNA: Deoxyribonucleic acid
MAR: Multiple antibiotic resistance index
MDR: Multidrug Resistant
NCBI: National Center for Biotechnology Information
PCR: Polymerase chain reaction
PDR: Pandrug resistant
RNA: Ribonucleic acid
SXT: Trimethoprim**/**sulfamethoxazole
XDR: Extensive Drug resistant
